# Narrowband Resting-State fNIRS Functional Connectivity in Autism Spectrum Disorder

**DOI:** 10.3389/fnhum.2021.643410

**Published:** 2021-06-15

**Authors:** Weiting Sun, Xiaoyin Wu, Tingzhen Zhang, Fang Lin, Huiwen Sun, Jun Li

**Affiliations:** ^1^South China Academy of Advanced Optoelectronics, South China Normal University, Guangzhou, China; ^2^Key Lab for Behavioral Economic Science & Technology, South China Normal University, Guangzhou, China

**Keywords:** functional near-infrared spectroscopy, autism spectrum disorder, resting-state functional connectivity, narrow frequency bands, discrimination

## Abstract

Hemispheric asymmetry in the power spectrum of low-frequency spontaneous hemodynamic fluctuations has been previously observed in autism spectrum disorder (ASD). This observation may imply a specific narrow-frequency band in which individuals with ASD could show more significant alteration in resting-state functional connectivity (RSFC). To test this assumption, we evaluated narrowband RSFC at several frequencies for functional near-infrared spectroscopy signals recorded from the bilateral temporal lobes on 25 children with ASD and 22 typically developing (TD) children. In several narrow-frequency bands, we observed altered interhemispheric RSFC in ASD. However, in the band of 0.01–0.02 Hz, more mirrored channel pairs (or cortical sites) showed significantly weaker RSFC in the ASD group. Receiver operating characteristic analysis further demonstrated that RSFC in the narrowband of 0.01–0.02 Hz might have better differentiation ability between the ASD and TD groups. This may indicate that the narrowband RSFC could serve as a characteristic for the prediction of ASD.

## Introduction

Over decades, the prevalence of autism spectrum disorder (ASD) has an astonishing rise in children (Goldson, 2016). It has been acknowledged that genetic, developmental, and environmental factors could be related to the effects toward developing autism (Arbabshirani et al., 2017). Individuals in the spectrum often present with symptoms such as impairments in social interaction and communication, restricted interests, and repetitive patterns of behaviors (Veenstra-VanderWeele et al., 2004). These symptoms generally appear in the first 2–3 years of life, and thereafter ASD can be reliably diagnosed based on the observation of these children's behaviors (Lord et al., 2006), e.g., *via* the Autism Diagnostic Observation Schedule (Luyster et al., 2009). Although ASD can be a lifelong disorder, early intervention for ASD is still extremely important, as proper care and function training can improve individuals' condition and help them learn new skills and find their places in the world as happy and productive human beings. For this reason, the earlier ASD is diagnosed, the sooner the intervention can be started.

Despite rapid progress on the study of ASD, the fact is that the diagnosis still relies solely on the behavioral observation, as to date there is no reliable biological indicator directly related to the disorder. Children with suspected ASD cannot receive a final diagnosis until they are much older, e.g., 2–3 years old, as only by then the consistent behaviors can be observed. This delay often prevents children with ASD from getting necessary and timely help.

In order to improve this situation, a great number of imaging studies for revealing intrinsic characteristics in the brains of ASD have been performed. Among these studies, cortical temporal correlation of the spontaneous brain activity in distinct but functionally related regions, termed as resting-state functional connectivity (RSFC), has become a research focus (Biswal et al., 1995; White et al., 2009; Lu et al., 2010; Van Den Heuvel and Pol, 2010; Zhang et al., 2010; Chuang and Sun, 2014; Li and Qiu, 2014; Smitha et al., 2017), because RSFC reveals concurrent spontaneous activity of spatially segregated regions (Friston, 2007). Studying RSFC may generate fresh insight into understanding of ASD, as well as other neurological and psychiatric disorders. Functional magnetic resonance imaging (fMRI) (Monk et al., 2009; Assaf et al., 2010) studies have demonstrated the existence of abnormalities in the functional connection in the brains of ASD. However, it is usually difficult to measure brain activity for young children because of their inability to remain still for several minutes, e.g., 5–10 min, a typical time duration required for studying resting state by using brain imaging modalities such as MRI/fMRI, positron emission tomography (PET), and magnetoencephalography (MEG). These brain imaging modalities usually require the subject to keep motionless during measurement. However, for functional near-infrared spectroscopy (fNIRS) it might not be a severe problem. It is because as compared to the aforementioned imaging techniques, fNIRS is more tolerant to motion artifact (MA) caused by head movement; thus, it is more suitable on studying children's brains. Besides this, the unrealistic environment required for fMRI, PET, and MEG measurement may cause claustrophobia, which may induce extra unexpected effect on the resting-state data. In addition, several fNIRS studies on ASD have already shown that children with ASD present atypical cortical activity (Kita et al., 2011), even in resting state (Zhu et al., 2014; Li et al., 2016; Xu et al., 2019, 2020a,b,c).

Resting-state fNIRS studies on ASD can be roughly divided into two categories: one is uncovering various characteristics associated with ASD in a single or several fNIRS channels (e.g., Xu et al., 2019, 2020a,b,c), and the other is investigating the difference between ASD and TD in the interhemispheric RSFC (e.g., Zhu et al., 2014; Li et al., 2016). The two categories of studies reveal resting-state features associated with ASD from different aspects, e.g., one is from a single or a few cortical sites and the other from brain network.

Previous studies have demonstrated that as compared to typically developing (TD) children, children with ASD show weaker RSFC between the bilateral temporal lobes (Zhu et al., 2014; Li et al., 2016) and asymmetry between the left and right hemispheres in the power spectrum of the low-frequency hemodynamic fluctuations. Moreover, the deviation in the power spectrum occurred mostly in the frequency band of 0.01–0.06 Hz (Cheng et al., 2019). In an fNIRS study, Sasai et al. found frequency-specific RSFC, implying that the correlation of spontaneous brain activity between different regions depended also on the frequency band (Sasai et al., 2011). In several fMRI studies, altered oscillation in Slow-5 band (0.010–0.027 Hz) was found in healthy aging adults and ischemic stroke patients, which was believed to be associated with the disruptions on the brain network (La et al., 2016a,b,c). Cheng et al. (2017) have found that dynamic functional connectivity (i.e., the temporal variation in RSFC) in Slow-5 band reflects the capacity of sustaining attention in performing a cognitive task during pain. As children with ASD have weaker interhemispheric brain network and usually show deficit in sustaining attention, it might be reasonable to infer their brain spontaneous fluctuations, and functional connectivity may also show pronounced alteration in Slow-5 band. Therefore, we assume that the most alteration in the functional connectivity between ASD and TD may occur in a specific narrow-frequency band, rather than in a broad low-frequency band, e.g., 0.009–0.08 Hz (hereafter referred to as broadband). To test this hypothesis and locate precisely a specific narrow-frequency band in which RSFC has a higher discriminative ability between ASD and TD, the fNIRS data collected in a prior study (Li et al., 2016) were reanalyzed. The frequency range from 0.01 to 0.06 Hz was focused on. The reason why we focused on this frequency range was because of our previous work (Cheng et al., 2019), in which we analyzed power spectrum of resting-state fNIRS signals recorded from bilateral temporal lobes on children with ASD and normal TD controls. We found within the broadband range that the significant alteration occurred mostly in 0.01–0.06 Hz in ASD, and also in this range, children with ASD exhibited an asymmetry between right and left brain hemispheres in the power spectrum. This asymmetry implies children with ASD may show an atypical homotopic RSFC within the range of 0.01–0.06 Hz. To find a specific bandwidth in which RSFC shows the most pronounced alteration in ASD, several narrow bandwidths including 0.01, 0.02, and 0.03 Hz were used for bandpass filter. After bandpass filtering, an independent component analysis (ICA) algorithm was applied to remove MA and suppress global component in the fNIRS signals. Narrowband RSFC was then obtained at each frequency band by calculating Pearson correlation coefficient between each mirrored channel pair. To demonstrate the significant difference between the ASD and TD groups in the narrowband RSFC, statistical hypothesis test and power analysis were performed. To further show the discrimination ability of the narrowband RSFC between the two groups, receiver operating characteristic (ROC) curve and area under curve (AUC) value were presented.

## Materials and Methods

### Participants

Twenty-five children with ASD (18 boys and 7 girls, 9.3 ± 1.4 years old) and 22 age-matched TD children (18 boys and 4 girls, 9.5 ± 1.6 years old) participated in the study. The children with ASD were recruited from a local autism rehabilitation school. They were all diagnosed by experienced pediatricians and child psychiatrists in hospitals on the basis of *Diagnostic and Statistical Manual of Mental Disorders, Fourth Edition, Text Revision* (American Psychiatry Association, 2000). The TD children were recruited from a local elementary school. Intelligence quotient (IQ) for both groups were measured with Raven's Standard Progressive Matrices Test (Raven and Court, 1998). IQ was 91 ± 15 for the ASD group and 106 ± 12 for the TD group. IQ difference between the two groups was significant (two-sample *t*-test: *p* < 0.05). Ethical approval was obtained from South China Normal University's Ethical Review Board, and informed consents were obtained from all participants' parents prior to the experiments.

### Data Acquisition

**Figure 1:**
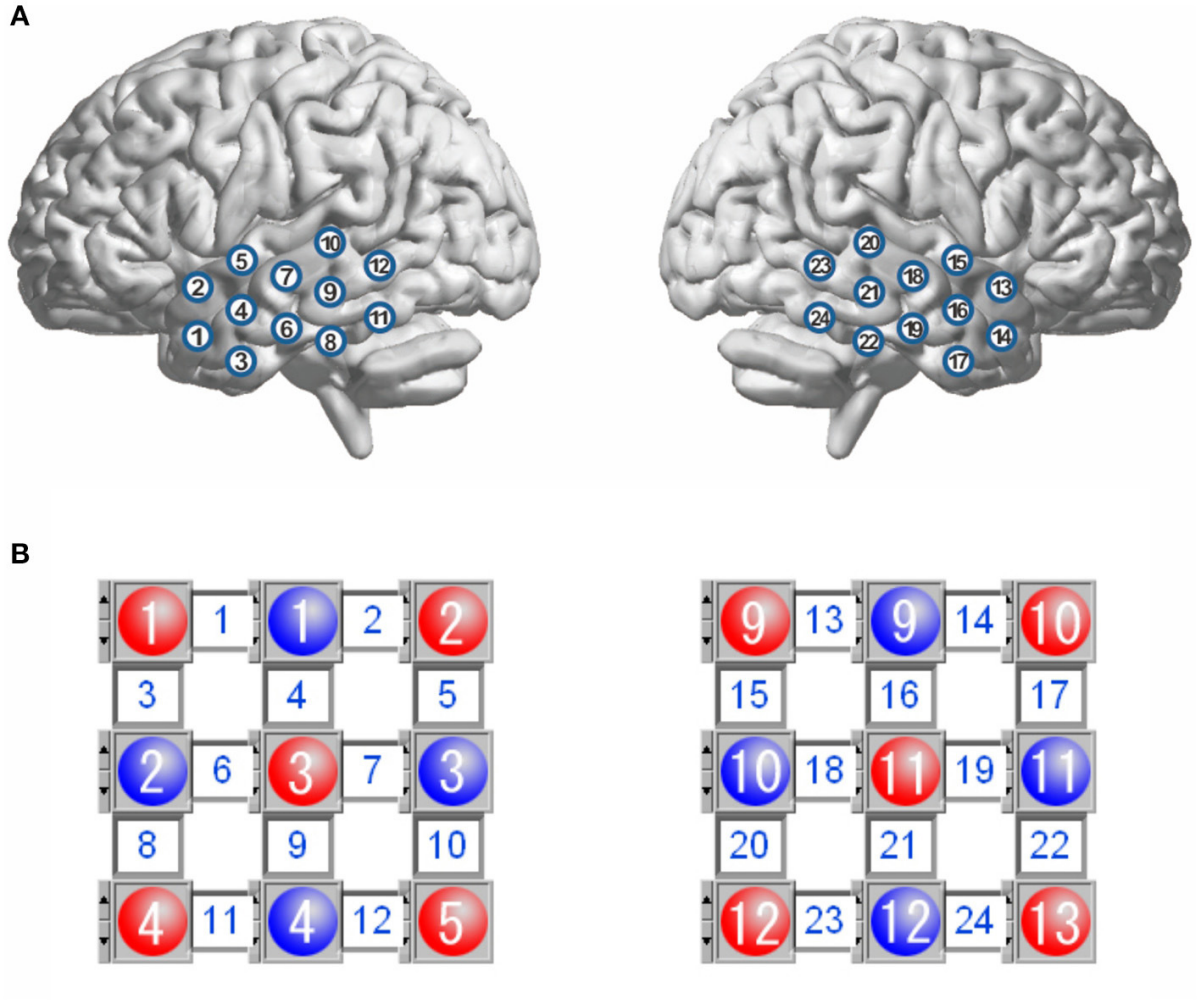
Schematic representation of channel locations over the bilateral temporal lobes **(A)**, and layout for the sources and detectors **(B)**. The red circles represent the sources, and the blue circles represent the detectors, and the square between a source and a detector is a channel. There are 12 channels in each hemisphere.

During resting-state fNIRS data acquisition, all participants were awake with their eyes closed and sat comfortably in a dark room. They were asked to try to keep as motionless as possible. The fNIRS used in this study was a commercial continuous-wave fNIRS system (FOIRE 3000, Shimadzu Corporation, Japan). To ensure a good optode-scalp contact, all optodes were secured on the scalp by a headgear. Twenty-four optical channels (a channel consisted of a source-detector pair) were used with 12 channels on each side of the head (Figure 1). The source-detector distance was fixed to be 3.0 cm. For each subject, spontaneous fluctuations of cortical oxygenated hemoglobin (HbO_2_), deoxygenated Hb, and total Hb (HbT = HbO_2_ + Hb) were recorded from the bilateral temporal lobes for about 8 min with 70-ms temporal resolution (i.e., 14.3-Hz sampling frequency). The output parameters of this fNIRS were concentration changes in HbO_2_, Hb, and HbT, which were converted from changes in optical density of three wave lengths (780, 805, and 830 nm) based on the modified Beer–Lambert law. The international 10/20 system was referenced for identifying channel locations on the bilateral temporal lobes.

### Data Analysis

The raw data we collected included HbO_2_, Hb, and HbT, where HbT is the sum of HbO_2_ and Hb. As overlapping information can hardly make a better contribution for the discrimination between ASD and TD, only HbO_2_ and Hb were analyzed. Each time series of the hemodynamic signal S (HbO_2_ and Hb) was first transformed into its *Z*-score *via Z* = (*S* – mean (S))/std (S), and then a detrending process with a second-order polynomial fit was applied to *Z*-score to remove slow drift. To select different frequency components for computing frequency-dependent RSFC, a zero-phase second-order Butterworth bandpass filter was applied with a variety of pass-bands (Table 1). As the upper limit of these pass-bands was <0.08 Hz, most systemic interferences were filtered out, such as those originated from cardiac cycles (~1 Hz), respirations (~0.2 Hz), and Mayer waves (~0.1 Hz). After the filtering, an ICA algorithm was employed to suppress global component and remove MA. In this ICA algorithm, we assumed that the global component would make similar contribution to all channels, which could be identified from the mixing matrix, whereas an MA could be an independent component whose peak value was 10 times larger than the standard deviation of this component. For more details on the ICA algorithm, readers can refer to Li et al. (2016).

**Table 1:** The number of channel pairs with significant difference in RSFC between ASD and TD in each frequency band for HbO_2_ and Hb, respectively.

**Frequency band (FB)**		**HbO_**2**_**	**Hb**
FB1	0.01–0.02 Hz	6	2
FB2	0.02–0.03 Hz	0	0
FB3	0.03–0.04 Hz	0	0
FB4	0.04–0.05 Hz	1	1
FB5	0.05–0.06 Hz	0	0
FB6	0.01–0.03 Hz	3	1
FB7	0.02–0.04 Hz	1	0
FB8	0.03–0.05 Hz	0	0
FB9	0.04–0.06 Hz	1	1
FB10	0.01–0.04 Hz	2	1
FB11	0.02–0.05 Hz	1	0
FB12	0.03–0.06 Hz	0	0
Broadband	0.009–0.08 Hz	2	0
Slow-5	0.010–0.027 Hz	3	1

After detrending, bandpass filtering, and ICA processing, narrowband hemodynamic signals were obtained for all optical channels. These signals were then used to compute interhemispheric RSFC for each subject. The RSFC was indicated *via* the Pearson correlation coefficient of the hemodynamic fluctuations for each mirrored channel pair locating symmetrically on the left and right hemispheres. As there were 12 optical channels on each hemisphere, 12 correlation coefficients were obtained for each subject. To test whether the difference in RSFC was significant between the two groups (ASD vs. TD) in each narrow-frequency band, two-sample *t*-test was first performed. Because of non-Gaussianity of the correlation coefficients, before performing *t*-test, each correlation coefficient was transformed into Fisher *Z* value by Fisher *Z* transform. For multiple comparisons, the false discovery rate (FDR) correction (i.e., FDR-corrected *q* value) was utilized. To control type II error, the statistical powers were also calculated for the differences with the current sample size. We defined significant difference in this study as both FDR-corrected *q* < 0.05 and power > 0.8. To further show the discrimination ability of RSFC in narrowbands, ROC curves and AUC values were presented.

## Results

RSFC was indicated as a Pearson correlation coefficient for each mirrored channel pair (Figure 1, 12 channel pairs in total) in hemodynamic signals (HbO_2_ and Hb). For all frequency bands, including 12 narrowbands (FB1–FB12), the broadband, and Slow-5 band, we counted the number of the mirrored channel pairs for which the correlation coefficients (in HbO_2_ and Hb) showed significant difference (defined as FDR-corrected *q* < 0.05 and statistical power >0.8) between the ASD and TD groups. The number in each band is listed in Table 1.

In FB1 frequency band (0.01–0.02 Hz), there were eight correlation coefficients (six in HbO_2_ and two in Hb) showing significant difference between the ASD and TD groups, more than those in the other frequency bands including the broadband (0.009–0.08 Hz) and Slow-5 band (0.010–0.027 Hz). In the broadband, only two correlation coefficients in HbO_2_ were observed to show significant difference between the two groups; whereas in Slow-5 band, three correlation coefficients in HbO_2_ and one in Hb showed significant difference, same as the number in FB6 (0.01–0.03 Hz), which almost fully overlay with the frequency range of Slow-5.

**Figure 2:**
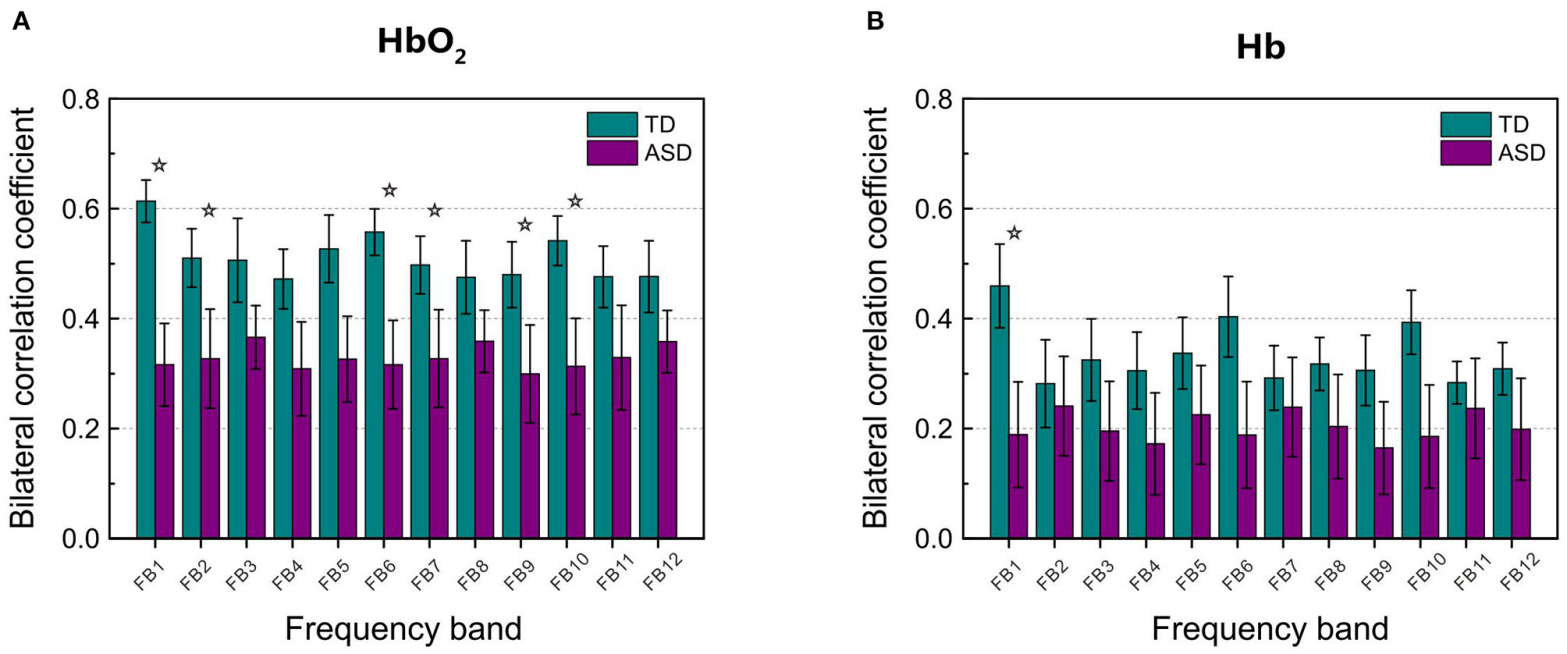
The grand average of the correlation coefficients for HbO_2_ **(A)** and Hb **(B)** between the left and right hemisphere in each frequency band (from FB1 to FB12, in total 12 bands) for TD and ASD groups. The asterisks indicate that the differences in the correlation coefficient between ASD and TD are statistically significant (FDR-corrected *q* < 0.05), and the statistical powers are larger than 0.8. FDR-corrected *q* values on HbO_2_ are 0.0024, 0.0048, 0.0032, 0.0106, 0.0144, and 0.0048 for FB1, FB2, FB6, FB7, FB9, and FB10, respectively. FDR-corrected *q* value on Hb is 0.0116 for FB1. The error bar for each correlation coefficient is the standard error of mean.

Figure 2 displays the grand average of correlation coefficients for the 12 (FB1–FB12) frequency bands. Two-sample *t*-test with FDR correction showed that the RSFC for both HbO_2_ and Hb in the frequency band of 0.01–0.02 Hz (FB1) had the smallest FDR-corrected *q* values: *q*(HbO_2_) = 0.0024, *q*(Hb) = 0.0116, indicating that in the band of 0.01–0.02 Hz, the homotopic RSFC for both HbO_2_ and Hb showed more pronounced difference between the ASD and TD groups. In addition, the power analysis showed with the current sample size (ASD = 25, TD = 22) that the difference (more strictly speaking the effect size) resulted in the power of 0.99 for HbO_2_ and 0.87 for Hb, both larger than 0.80, implying the current sample size was suitable for obtaining the conclusion that the difference between the two groups was statistically significant. As in the FB1 band, both the grand average of RSFC (Figure 2) and the group average of RSFC for mirrored channels (or cortical sites) (Table 1) showed more pronounced differences between the two groups, indicating that in the frequency band of 0.01–0.02 Hz, asynchronization in the spontaneous fluctuations between the two hemispheres was more obvious in ASD; thereafter, we focused on FB1 band for the further analysis.

**Figure 3:**
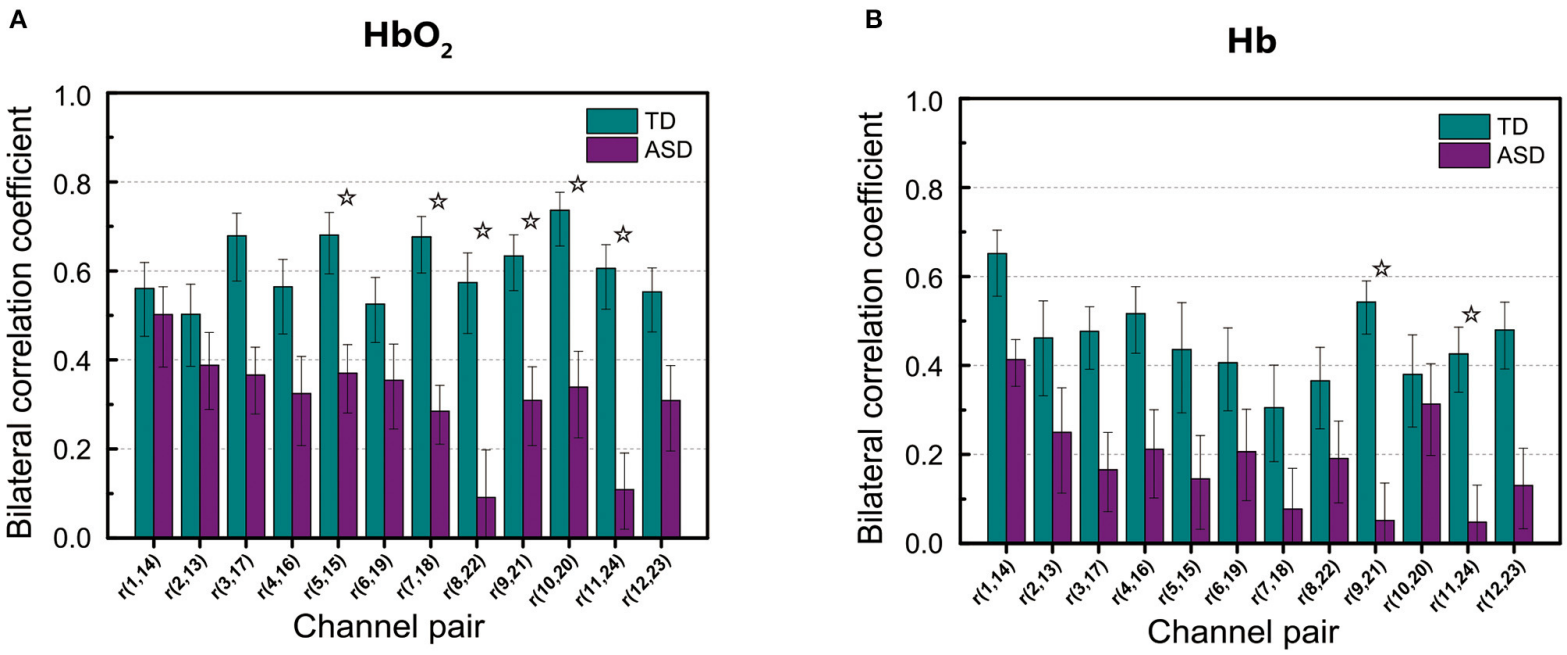
The group average of correlation coefficients of HbO_2_ **(A)** and Hb **(B)** in FB1 band for each mirrored channel pair located symmetrically on the left and right temporal lobes. The digit pair in the parenthesis indicates the corresponding channel pair, e.g., *r*(1,14) is the temporal correlation coefficient between channel 1 and channel 14. The asterisks indicate the differences in the correlation coefficient between ASD and TD are statistically significant (FDR-corrected *q* < 0.05) for the channel pair, and the statistical powers are larger than 0.8. The error bar for each correlation coefficient is the standard error of mean.

Figure 3 shows RSFC in HbO_2_ and Hb for the 12 mirrored channel pairs in FB1 band. As Figure 3 illustrates, children with ASD showed clearly weaker interhemispheric RSFC than TD children in both HbO_2_ and Hb, particularly between Ch5 and 15, Ch7 and 18, Ch8 and 22, Ch9 and 21, Ch10 and 20, and Ch11 and 24 in HbO_2_, and between Ch9 and 21, and Ch11 and 24 in Hb. For these channel pairs, the differences in the correlation coefficients were statistically significant, indicating the disruption in the synchronization of the spontaneous fluctuations in these mirrored cortical regions occurred more pronouncedly in the band of 0.01–0.02 Hz in autism brains.

**Figure 4:**
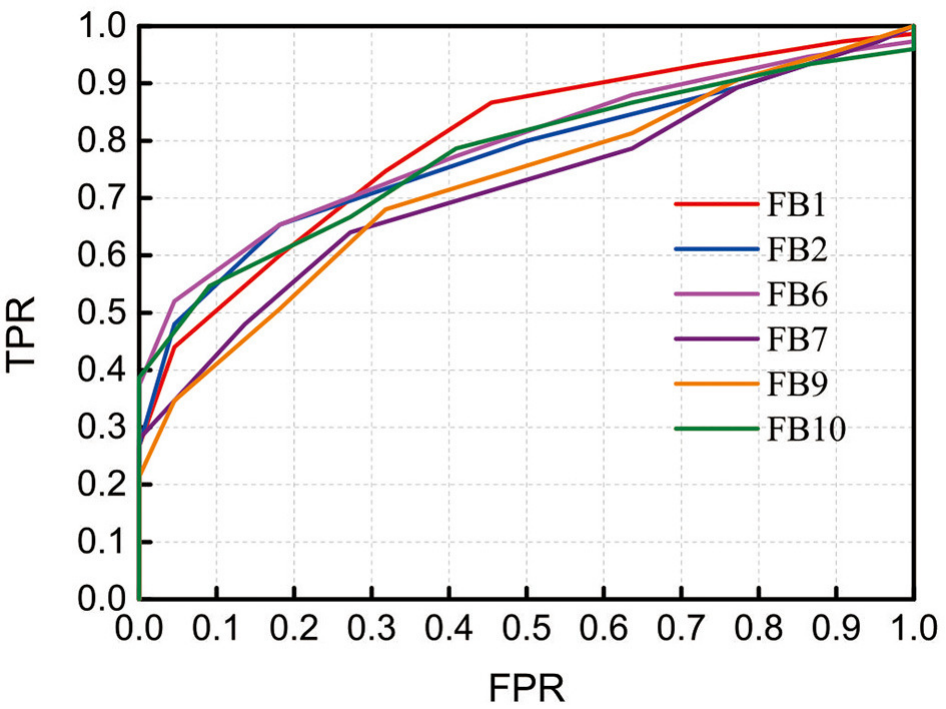
Receiver operating characteristic (ROC) curves obtained by using RSFC as discriminative features at several frequency bands in which RSFC is significantly different between the ASD and TD groups. The RSFC in each band is the mean of RSFC averaged over all channel pairs. AUC (FB1) = 0.806, AUC (FB2) = 0.783, AUC (FB6) = 0.797, AUC (FB9) = 0.734, and AUC (FB10) = 0.783. FPR, false-positive rate; TPR, true-positive rate.

To demonstrate the discrimination ability of FB1-RSFC between ASD and TD, we took the mean of all 12 RSFCs of each hemodynamic variable as a discriminative feature to draw ROC curves, as shown in Figure 4, for FB1 and the other bands in which the grand average of RSFC also showed a significant difference between the ASD and TD groups, for example, FB2, FB6, FB7, FB9, and FB10 (Figure 2). The ROC curve was generated by varying a criterion value for RSFC. An individual whose HbO_2_-RSFC and Hb-RSFC both were smaller than the criterion was classified as ASD, while classified as TD. The AUC value was calculated for each of these frequency bands: AUC (FB1) = 0.806, AUC (FB2) = 0.783, AUC (FB6) = 0.797, AUC (FB9) = 0.734, and AUC (FB10) = 0.783. AUC of FB1 was the largest among these bands, indicating FB1-RSFC is superior to RSFC in other frequency bands on the discrimination ability.

**Figure 5:**
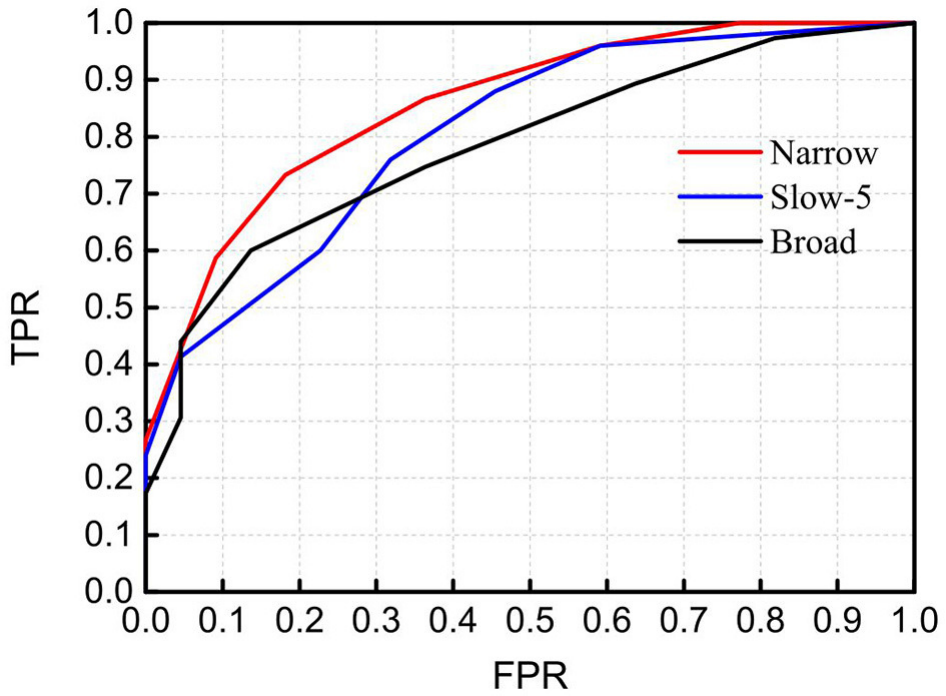
Receiver operating characteristic (ROC) curves obtained by using RSFC as discriminative features in the narrowband of 0.01–0.02 Hz (Narrow), Slow-5 band (Slow-5), and broadband of 0.009–0.08 Hz (Broad). The RSFC for each band is the mean of RSFC averaged over channel pairs showing significantly different connectivity between the ASD and TD groups. AUC (Narrow) = 0.867, AUC (Slow-5) = 0.817, and AUC (Broad) = 0.793. FPR, false-positive rate; TPR, true-positive rate.

As not all mirrored channel pairs in FB1-RSFC showed significant difference between the ASD and TD groups, to enhance the discrimination ability, we could calculate an average only over those channel pairs showing significant difference and took this average as a discriminative feature. To show the discriminative ability of this averaged FB1-RSFC, ROC curve was generated, as shown in Figure 5. For comparisons, ROC curves were also generated by using the averaged Slow-5 and broadband RSFC as discriminative feature. For these two bands, the average of RSFC was also calculated only over those channel pairs showing significant difference between the ASD and TD groups. The AUC value for the narrowband (FB1) was 0.867, larger than that using the averaged RSFC over all channel pairs (i.e., 0.806). This was because including the RSFC showing no significant difference between ASD and TD as discriminative feature could not make positive contribution to the discrimination, but could lead to some degradation. The AUC value was 0.817 for Slow-5 and 0.793 for the broadband, both smaller than the AUC value for the narrow FB1 band.

## Discussion

As FB6 (0.01–0.03 Hz) and FB10 (0.01–0.04 Hz) fully cover FB1 (0.01–0.02 Hz) in frequency range, it is not surprising that their AUC values are similar, e.g., 0.806, 0.797, and 0.783 for FB1, FB6, and FB10, respectively (Figure 4). However, from FB1 to FB6 and FB10, AUC shows a decreasing trend, which may imply that including those components in the frequency range beyond FB1 (such as 0.02–0.03 Hz and 0.02–0.04 Hz) cannot make better contribution to the differentiation than using FB1 only. The possible reason would be those bands provide either no useful information for the discrimination or redundant (e.g., overlapping) information with respect to FB1. To clarify this, we investigated the relationship between RSFCs in FB1, FB2 (0.02–0.03 Hz), FB7 (0.02–0.04 Hz), FB6, and FB10 by calculating the correlation coefficients between each other. The correlation coefficient was 0.733 between FB1- and FB2-RSFC, 0.722 between FB1- and FB7-RSFC, 0.973 between FB1- and FB6-RSFC, and 0.965 between FB1- and FB10-RSFC. As the correlation between FB1- and FB2-RSFC (also between FB1- and FB7-RSFC) was high (e.g., >0.7), RSFC in FB2 (0.02–0.03 Hz) and FB7 (0.02–0.04 Hz) could not provide additional discriminative information than FB1-RSFC. This could also be verified by the very high correlation between BF1- and FB6-RSFC (i.e., 0.973) and between BF1- and FB10-RSFC (i.e., 0.965). This correlation analysis could demonstrate that the most useful discriminative information was contained in FB1 band.

In an autism study with fMRI, Dinstein et al. (2011) observed the reduced interhemispheric fMRI-RSFC in autism, in particular RSFC between the bilateral superior temporal gyrus. To make a classification between all the participants with ASD (the number was 29) and all non-autistic controls (the number was 43), they selected a criterion value of 0.38 for the RSFC. With this criterion, all participants were classified as two categories: those with lower RSFC than the criterion were classified as ASD, otherwise classified as TD. This method yielded a classification with a sensitivity of 72.4%, specificity of 83.7%, and accuracy of 79.2%. As both the present and Dinstein and colleagues' studies used the homotopic RSFC in temporal lobes as a classification feature, to make a quantitative comparison on the classification ability between the fNIRS and fMRI data, we could use the same approach by selecting a criterion value on fNIRS-RSFC for best differentiating between the two groups. The result showed that by using the mean of RSFC showing significant difference between ASD and TD in FB1 band as discriminative feature and selecting a criterion value of 0.53, the classification could be achieved with a sensitivity of 80.0%, specificity of 81.8%, and accuracy of 80.9%, slightly better in sensitivity and accuracy than the classification obtained with fMRI. However, because of the difference in sample size (in Dinstein and colleagues' work, ASD = 29, non-ASD = 43, whereas in our study, ASD = 25, non-ASD = 22) and individuals, we could not conclude that fNIRS was superior to fMRI in RSFC-based classification between individuals with ASD and TD controls. Nevertheless, the discrimination ability of fNIRS is comparable to fMRI, demonstrating the promise of this optical imaging technique in the application of ASD study.

**Figure 6:**
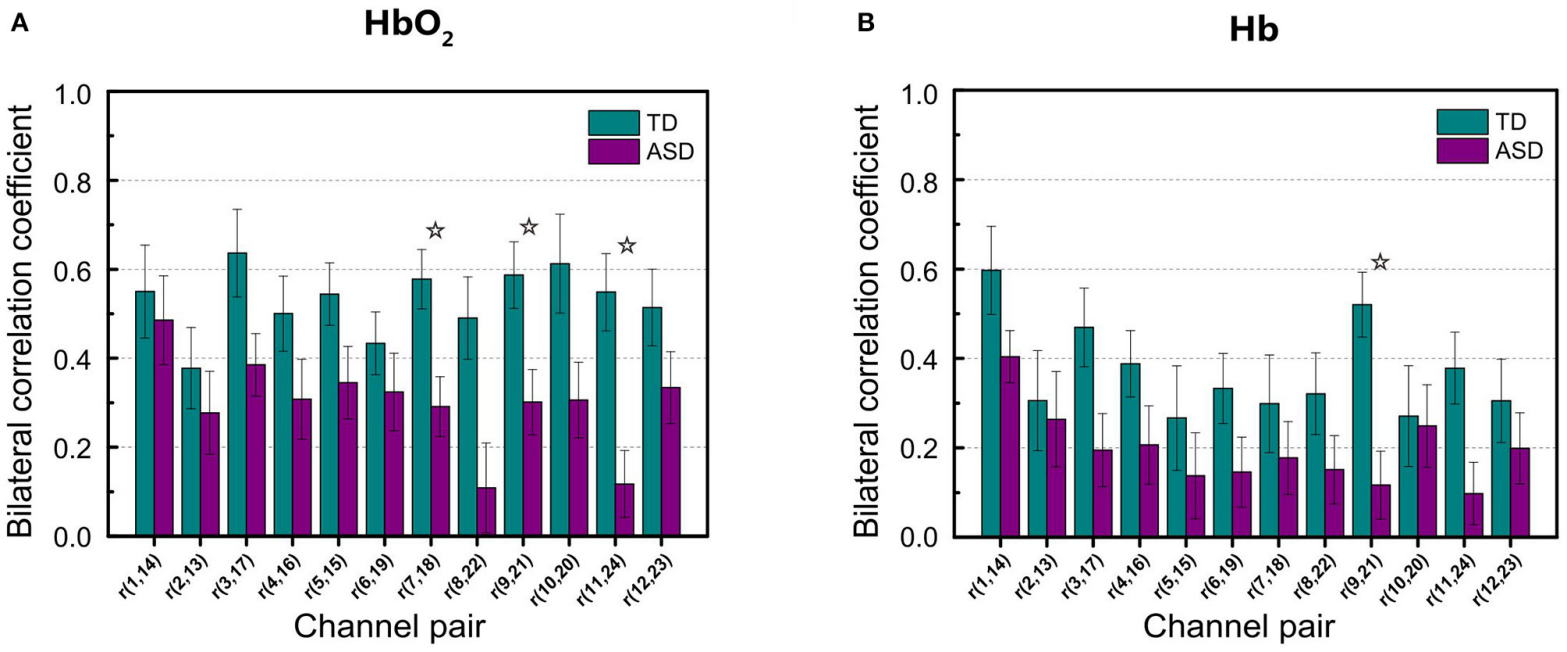
Group average of the correlation coefficients in HbO_2_ **(A)** and Hb **(B)** for each mirrored channel pair locating symmetrically on the left and right temporal lobes in Slow-5 band. The digit pair in the parenthesis indicates the corresponding channel pair, e.g., *r*(1,14) is the temporal correlation coefficient between channel 1 and channel 14. The asterisks indicate the differences in the correlation coefficient between ASD and TD are statistically significant (FDR-corrected *q* < 0.05) for the channel pair, and the statistical powers are larger than 0.8. The error bar for each correlation coefficient is the standard error of mean.

As seen in Table 1, in the narrow-frequency band of 0.01–0.02 Hz (FB1), the number of channel pairs showing significant differences in RSFC between ASD and TD is 8 (i.e., 6 in HbO_2_ and 2 in Hb), the largest number in all these frequency bands. In 0.01–0.03 Hz (FB6), the number is 4 (3 in HbO_2_, 1 in Hb), the second largest number. This frequency band mostly overlaps with Slow-5 band (0.010–0.027 Hz). As already discussed in the introduction, the reduced brain network connectivity could be manifested by the alteration in Slow-5 oscillation (La et al., 2016a,b,c); we further investigated if children with ASD could show altered Slow-5 RSFC. Figure 6 shows Slow-5 RSFC for each mirrored channel pair in TD and ASD groups. Not surprisingly, in Slow-5 band, there were three channel pairs, i.e., *r*(7,18), *r*(9,21), and *r*(11,24) in HbO_2_ and one channel pair, i.e., *r*(9,21), in Hb showing significant difference between the ASD and TD groups in the correlation coefficient, the same as seen in FB6 (0.01–0.03 Hz) (Table 1), and all pairs were included in those channel pairs observed in FB1 (Figure 3). As in the present study the minimal resolution of the frequency scanning was 0.1 Hz, the characteristics in Slow-5 might be reflected in FB1 (0.01–0.02 Hz) and FB6 (0.01–0.03 Hz), which could also be seen in Figures 3, 5, 6 and Table 1. From the ROC curve and AUC value, we could roughly evaluate the discrimination ability of RSFC between ASD and TD at the three frequency bands: 0.01–0.02 Hz (Narrow FB1), 0.010–0.027 Hz (Slow-5), and 0.009–0.08 Hz (broadband): narrow FB1 > Slow-5 > broadband (Figure 5).

In the present study, we observed in several frequency bands including FB1, FB2, FB6, FB7, FB9, and FB10 that the grand average of RSFC in HbO_2_ showed significant difference between the ASD and TD groups (Figure 2). However, the discrimination ability was different, as AUC value showed FB1-RSFC was superior to RSFC in the other frequency bands (Figure 4). In addition, correlation analysis demonstrated that RSFC in the other frequency bands was highly correlated to FB1-RSFC. Therefore, FB1-RSFC might contain more rich discriminative information than RSFC in the other bands. Besides that, only in the FB1 band that the grand average of RSFC in Hb showed significant difference between the ASD and TD groups. On the other hand, from the number of channel pairs (corresponding to the mirrored cortical sites) showing significant difference in RSFC, we found more mirrored cortical sites exhibited significantly weaker RSFC in ASD in the narrowband of FB1. Therefore, our fNIRS data and analysis suggested that FB1 (0.01–0.02 Hz) might be an important frequency band for studying atypical RSFC in ASD.

fNIRS, as a rapidly developing brain imaging technique, allows mapping human brain non-invasively. Its unique and beneficial characteristics, in particular relatively low sensitivity to head movement and natural environment for measurement, have made fNIRS gain more popularity in recent years, particularly in studying brains of young children. The discrimination ability between ASD and TD with fNIRS 0.01–0.02-Hz frequency band RSFC is comparable to fMRI-RSFC, making this optical imaging modality a potential tool for the prediction of ASD in children. Suffice to say, it might be of great significance to promote fNIRS with more clinical applications on ASD.

As the sample size (i.e., 25 children with ASD and 22 TD children) was limited in the present study, to effectively restrain type II error, we also performed statistical power analysis for the differences already passing through *t*-test and FDR correction. We defined significant difference by using two criteria: FDR-corrected *q* < 0.05 and statistical power >0.8. By this definition, both type I error (controlled by *t*-test and FDR correction) and type II error (controlled by the statistical power analysis) would be strictly restrained; thus, the results observed were more statistically reliable, implying the revealed discriminative features should likely be still valid when the sample (children with ASD and TD children) size is enlarged. Another limitation of this study is that we cannot completely exclude the possibility that the difference in RSFC is due to the difference in IQ between the two groups, although several fMRI studies (Song et al., 2008; Van Den Heuvel et al., 2009; Pamplona et al., 2015) on whole head have revealed that IQ is correlated to the functional connectivity between the frontal and other brain regions such as parietal, occipital, and limbic lobes, but have not reported any evidence that IQ is related to RSFC between the bilateral temporal lobes.

## Conclusion

We applied narrowband pass filters with different bandwidths to resting-state fNIRS data and found that children with ASD showed significantly weaker RSFC than TD children in a variety of frequency bands. However, the difference between ASD and TD in RSFC was more pronounced in 0.01–0.02 Hz than the other frequency bands including the widely used broadband (0.009–0.08 Hz) and Slow-5 band. ROC curve and AUC value also demonstrated 0.01–0.02-Hz RSFC could be a good characteristic feature for the discrimination between children with ASD and TD children.

## Data Availability Statement

The raw data supporting the conclusions of this article will be made available by the authors, without undue reservation.

## Ethics Statement

The studies involving human participants were reviewed and approved by South China Normal University's Ethical Review Board. Written informed consent to participate in this study was provided by the participants' legal guardian/next of kin.

## Author Contributions

JL and WS conceptualized the manuscript. WS, XW, and TZ analyzed the data. WS wrote the original draft. HS and FL drew pictures. JL reviewed and edited its intellectual content. All authors contributed to the article and approved the submitted version.

## Conflict of Interest

The authors declare that the research was conducted in the absence of any commercial or financial relationships that could be construed as a potential conflict of interest.
